# The cocrystal 10-(2-methyl-4-phenyl-1*H*-inden-6-yl)-10*H*-phenothia­zine–10-(2-methyl-7-phenyl-1*H*-inden-5-yl)-10*H*-phenothia­zine (0.54/0.46)

**DOI:** 10.1107/S1600536808036210

**Published:** 2008-11-13

**Authors:** Mikhail V. Nikulin, Alexander Z. Voskoboynikov, Kyrill Yu. Suponitsky

**Affiliations:** a Moscow State University, Leninskie Gory, GSP-2, Moscow 119992, Russian Federation; b A.N. Nesmeyanov Institute of Organoelement Compounds, Russian Academy of Sciences, Vavilov St., 28, Moscow 119991, Russian Federation

## Abstract

The title compound, 0.535C_28_H_21_NS.0.465C_28_H_21_NS, was synthesized by palladium-catalysed amination. The structure is composed of two isomeric mol­ecules, *viz*. 10-(2-methyl-4-phenyl-1*H*-inden-6-yl)-10*H*-phenothia­zine, and 10-(2-methyl-7-phenyl-1*H*-inden-5-yl)-10*H*-phenothia­zine, in the refined ratio 0.535 (12):0.465 (12). The isomers differ by the localization of the double bond in the cyclo­penta­diene ring. There are two sites in the structure that are occupied by the isomers. The respective isomers are occupationally disordered in each site, the refined proportions being 0.640 (6):0.360 (6) and 0.43 (1):0.57 (1). Moreover, each isomeric mol­ecule is chiral; due to the crystallographic centres of symmetry, the mol­ecules are also present in enanti­omeric pairs. The crystal structure is stabilized by weak π–π [C⋯C = 3.389 (2) Å] inter­actions.

## Related literature

The title product was used to obtain the pertinent ansa-zirconocene for isotactic olefin polymerization study, see: Voskoboynikov *et al.* (2006). For a description of the Cambridge Structural Database, see: Allen (2002 ▶). For bond-lengths data, see: Allen *et al.* (1987 ▶). For details of the Buchwald–Hartwig amination protocol used in the synthesis, see: Yang & Buchwald (1999 ▶).
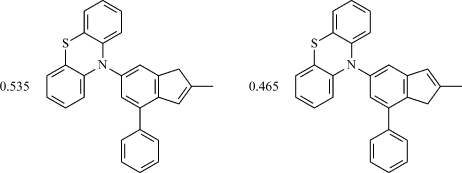


## Experimental

### 

#### Crystal data

0.535C_28_H_21_NS·0.465C_28_H_21_NS
*M* *_r_* = 403.52
Triclinic, 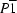

*a* = 12.6480 (6) Å
*b* = 12.6654 (6) Å
*c* = 14.6970 (7) Å
α = 77.4920 (10)°
β = 64.8070 (10)°
γ = 82.9690 (10)°
*V* = 2078.62 (17) Å^3^
*Z* = 4
Mo *K*α radiation
μ = 0.17 mm^−1^
*T* = 100 (2) K
0.20 × 0.15 × 0.10 mm

#### Data collection

Bruker SMART APEXII CCD diffractometer
Absorption correction: multi-scan (*APEX2*; Bruker, 2005 ▶) *T*_min_ = 0.961, *T*_max_ = 0.983
25507 measured reflections
10999 independent reflections
7713 reflections with *I* > 2σ(*I*)
*R*_int_ = 0.036

#### Refinement

*R*[*F* ^2^ > 2σ(*F* ^2^)] = 0.045
*wR*(*F* ^2^) = 0.105
*S* = 1.01
10999 reflections
569 parameters
44 restraints
H-atom parameters constrained
Δρ_max_ = 0.35 e Å^−3^
Δρ_min_ = −0.34 e Å^−3^

### 

Data collection: *APEX2* (Bruker, 2005 ▶); cell refinement: *SAINT* (Bruker, 2005 ▶); data reduction: *SAINT*; program(s) used to solve structure: *SHELXTL* (Sheldrick, 2008 ▶); program(s) used to refine structure: *SHELXTL*; molecular graphics: *SHELXTL*; software used to prepare material for publication: *SHELXTL*.

## Supplementary Material

Crystal structure: contains datablocks I, global. DOI: 10.1107/S1600536808036210/fb2115sup1.cif

Structure factors: contains datablocks I. DOI: 10.1107/S1600536808036210/fb2115Isup2.hkl

Additional supplementary materials:  crystallographic information; 3D view; checkCIF report

## Figures and Tables

**Fig. 1.**
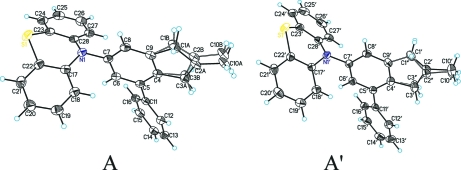
The view of the title molecules, showing the atom-numbering scheme and the displacement ellipsoids drawn at 50% probability level. The hydrogen atoms are shown as spheres of arbitrary radius. The major-occupied parts at the sites A and A' correspond to the isomers (Ia) and (Ib) respectively. The disorder is also shown for both sites A and A'. Bonding in the minor-occupied part is presented with open solid lines.

## supplementary crystallographic information

### Comment

The title product was used to obtain the pertinent ansa-zirconocene for isotactic olefin polymerization study (Voskoboynikov *et al.*, (YEAR?)). Herein we report the crystal structure which was synthesized by Buchwald–Hartwig amination protocol (Yang & Buchwald, 1999) from the mixture of the 6-chloro-2-methyl-4-phenyl-1*H*-indene and 5-chloro-2-methyl-7-phenyl-1*H*-indene, and 10*H*-phenothiazine in the presence of lithium *tert*-butoxide and Pd(P*^t^*Bu_3_)_2_ in toluene at reflux.

The title structure consists of two independent isomers (Fig. 1): 10-(2-methyl-4-phenyl-1*H*-inden-6-yl)-10*H*-phenothiazine (Ia), and 10-(2-methyl-7-phenyl-1*H*-inden-5-yl)-10*H*-phenothiazine (Ib). These molecules are situated in two sites (A and A') in the structure. In each site, the isomeric molecules are occupationally disordered. In the site A, the isomers (Ia) and (Ib) superimpose in the ratio of 0.640 (6):0.360 (6), respectively, while for the site A' the nearly reverse proportion 0.43 (1):0.57 (1) is observed. Therefore the total ratio of two isomers in the crystal structure is 0.535 (12):0.465 (12) which is in the reasonable agreement with the data of a ^1^H NMR study which showed 1:1 ratio.

In the title structure, all the bond lengths and angles show normal values (Allen *et al.*, 1987; Allen, 2002; CSD (2007), version 5.29). In both isomers, the phenothiazine fragment is bent relative to the line connecting S1(S1') and N1(N1') atoms; the corresponding interplanar angles equal to 147.72 (3) and 147.69 (4)° for A and A' site, respectively. In each isomer, the six-membered 1,4-thiazine ring adopts the boat conformation. In each isomer, the indene ring is nearly planar (the mean deviations are 0.025° and 0.043Å for A and A', respectively). The indene rings are nearly perpendicular to the mean planes of the phenothiazine fragments (the interplanar angles are 84.24 (12) and 86.49 (12)° for A and A', respectively). The phenyl substituent at C5 (C5') atoms is rotated out of the plane of the indene ring (the interplanar angles are 41.96 (13) and 42.8 (2)° for A and A', respectively). The relative orientations of the molecular fragments are plausibly influenced by the steric repulsions of the closest hydrogen atoms. The molecules at the site A' form dimers by means of weak π–π interactions (C4'···C15'(-*x*, 2-*y*, -*z*), 3.389 (2) Å).

### Experimental

Under argon atmosphere, a mixture of 9.63 g (40.0 mmol) of 6-chloro-2-methyl-4-phenyl-1*H*-indene and 5-chloro-2-methyl-7-phenyl-1*H*-indene, 9.60 g (120 mmol) of *^t^*BuOLi, 7.97 g (40.0 mmol) of 10*H*-phenothiazine, 460 mg (0.80 mmol) of Pd(dba)_2_, 320 mg (1.60 mmol) of tris(*tert*-butyl)phosphine, and 80 ml of toluene was refluxed for 10 h. The resulting mixture was cooled to room temperature, passed through a short column with silica gel 60 (40–63 µm, d 80 mm, l 50 mm). The silica gel layer was additionally washed by 300 ml of methyl*tert*-butyl ether. The combined elute was evaporated to dryness. The crude product was purified by flash-chromatography on silica gel 60 (40–63 µm, d 60 mm, l 500 mm; eluent: hexane/methyl*tert*-butyl ether 20:1, vol.). Yield 10.7 g (66%) of *approx.* 1:1 mixture of 10-(2-methyl-4-phenyl-1*H*-inden-6-yl)-10*H*-phenothiazine (the isomer (Ia)) and 10-(2-methyl-7-phenyl-1*H*-inden-5-yl)-10*H*-phenothiazine (the isomer (Ib)). The yellow single crystals suitable for the X-ray analysis (in the form of prisms and with the size of 0.20 × 0.15 × 0.10 mm) were grown from toluene after a few days.

^1^H NMR (CDCl_3_): δ 6.74–7.59 (m, 7-H in indenyl of the isomer (Ib) and 3,7- H in indenyl of the isomer (Ia), Ph and phenothiazine of the isomers (Ia) and (Ib)), 6.59 (m, 3-H in indenyl of the isomer (Ib)), 6.35 (d, J= 7.7 Hz, 5-H in indenyl of the isomer (Ib)), 6.32 (d, J= 7.7 Hz, 5-H in indenyl of the isomer (Ia)), 3.50 (s, 2H, CH_2_ of the isomer (Ib)), 3.46 (s, CH_2_ of the isomer (Ia)), 2.17 (m, 2-Me of the isomers (Ia) and (Ib)).

^13^C{^1^H} NMR (CDCl_3_): 6 149.0, 148.4, 148.3, 147.9, 146.5, 144.6, 144.5, 143.5, 140.6, 140.2, 139.7, 139.5, 136.7, 135.7, 129.4, 128.7, 128.5*, 128.3, 127.5, 127.2, 127.0, 126.8*, 126.5*, 126.0, 124.7, 122.2, 121.2*, 119.6, 115.87, 115.81, 43.1, 42.7, 17.0, 16.8 (* two resonances).

### Refinement

All the H atoms except some of them in the disordered region could be distinguished in the difference electron density map. However, they were situated into the idealized positions and refined in the riding motion approximation with C—H distances equal to 0.98Å for the methyl groups, 0.95Å for the aryl carbon atoms, and 0.99Å for the carbon atoms of the cyclopentadiene ring in the *sp*^3^ state. *U*_iso_(H)=1.5*U*_eq_(parent atom) for the methyls and *U*_iso_(H)=1.2*U*_eq_(parent atom) for the remaining atoms. For the methyl hydrogen atoms, AFIX 137 command was used (Sheldrick, 2008). In the A and A' sites, the molecules are occupatioanlly disordered, being a superposition of the isomers (Ia) and (Ib). The proportions of (Ia) to (Ib) in the sites A and A' were refined with isotropic thermal displacement parameters using EADP command to equalize thermal parameters for the corresponding atomic pairs: C1A and C1B; C1' and C1"; C2A and C2B; C2' and C2"; C3A and C3B; C3' and C3"; C10A and C1B; C10' C10". These proportions were fixed in further refinement. In the next step all the disordered carbon atoms were refined unisotropically. The disordered fragments of the sites A and A' were refined using the command SAME 0.005 0.005 for (C3A C2A C1A C10A), (C1B C2B C3B C10B), (C1' C2' C3' C10'), (C3" C2" C1" C10"). In addition, the following commands were used: DFIX 1.492 0.005 C9 C1A C4 C3B C4' C3' C9' C1"; DFIX 1.464 0.005 C4 C3A C9 C1B C9' C1' C4' C3". (The target distances were retrieved from the Cambridge Crystallographic Database (2007 Version; Allen, 2002). Moreover, the above mentioned EADP commands were also applied.

### Crystal data

**Table:**

0.535C_28_H_21_NS·0.465C_28_H_21_NS	*Z* = 4
*M**_r_* = 403.52	*F*_000_ = 848
Triclinic, *P*1	*D*_x_ = 1.289 Mg m^−^^3^
Hall symbol: -P 1	Mo *K*α radiation λ = 0.71073 Å
*a* = 12.6480 (6) Å	Cell parameters from 5458 reflections
*b* = 12.6654 (6) Å	θ = 2.4–28.8º
*c* = 14.6970 (7) Å	µ = 0.17 mm^−^^1^
α = 77.4920 (10)º	*T* = 100 (2) K
β = 64.8070 (10)º	Prism, yellow
γ = 82.9690 (10)º	0.20 × 0.15 × 0.10 mm
*V* = 2078.62 (17) Å^3^	

### Data collection

**Table:**

Bruker SMART APEXII CCD diffractometer	10999 independent reflections
Radiation source: fine-focus sealed tube	7713 reflections with *I* > 2σ(*I*)
Monochromator: graphite	*R*_int_ = 0.036
*T* = 100(2) K	θ_max_ = 29.0º
φ and ω scans	θ_min_ = 1.7º
Absorption correction: multi-scan(APEX2; Bruker, 2005)	*h* = −17→17
*T*_min_ = 0.961, *T*_max_ = 0.983	*k* = −17→17
25507 measured reflections	*l* = −20→20

### Refinement

**Table:**

Refinement on *F*^2^	Secondary atom site location: difference Fourier map
Least-squares matrix: full	Hydrogen site location: difference Fourier map
*R*[*F*^2^ > 2σ(*F*^2^)] = 0.045	H-atom parameters constrained
*wR*(*F*^2^) = 0.105	*w* = 1/[σ^2^(*F*_o_^2^) + (0.0417*P*)^2^ + 0.4032*P*] where *P* = (*F*_o_^2^ + 2*F*_c_^2^)/3
*S* = 1.01	(Δ/σ)_max_ < 0.001
10999 reflections	Δρ_max_ = 0.35 e Å^−^^3^
569 parameters	Δρ_min_ = −0.34 e Å^−^^3^
44 restraints	Extinction correction: none
Primary atom site location: structure-invariant direct methods	

### Special details

**Table:**

Geometry. All esds (except the esd in the dihedral angle between two l.s. planes) are estimated using the full covariance matrix. The cell esds are taken into account individually in the estimation of esds in distances, angles and torsion angles; correlations between esds in cell parameters are only used when they are defined by crystal symmetry. An approximate (isotropic) treatment of cell esds is used for estimating esds involving l.s. planes.
Refinement. Refinement of *F*^2^ against ALL reflections. The weighted *R*-factor *wR* and goodness of fit *S* are based on *F*^2^, conventional *R*-factors *R* are based on *F*, with *F* set to zero for negative *F*^2^. The threshold expression of *F*^2^ > σ(*F*^2^) is used only for calculating *R*-factors(gt) *etc*. and is not relevant to the choice of reflections for refinement. *R*-factors based on *F*^2^ are statistically about twice as large as those based on *F*, and *R*- factors based on ALL data will be even larger.

### Fractional atomic coordinates and isotropic or equivalent isotropic displacement parameters (Å^2^)

**Table:**

	*x*	*y*	*z*	*U*_iso_*/*U*_eq_	Occ. (<1)
S1	−0.38737 (4)	0.28779 (4)	0.38391 (4)	0.03348 (11)	
N1	−0.14054 (11)	0.36876 (10)	0.28600 (10)	0.0213 (3)	
C3A	0.3165 (4)	0.5267 (8)	0.0573 (4)	0.0219 (11)	0.64
H3AA	0.3691	0.5586	0.0735	0.026*	0.64
C2A	0.3355 (5)	0.5131 (4)	−0.0372 (3)	0.0231 (9)	0.64
C1A	0.2319 (5)	0.4617 (9)	−0.0324 (3)	0.0246 (11)	0.64
H1AA	0.2552	0.3920	−0.0569	0.030*	0.64
H1AB	0.1956	0.5100	−0.0746	0.030*	0.64
C10A	0.4377 (3)	0.5458 (3)	−0.1361 (3)	0.0311 (9)	0.64
H10A	0.4939	0.5814	−0.1241	0.047*	0.64
H10B	0.4110	0.5960	−0.1836	0.047*	0.64
H10C	0.4752	0.4814	−0.1656	0.047*	0.64
C1B	0.2296 (9)	0.4449 (17)	−0.0275 (5)	0.0246 (11)	0.36
H1BA	0.2144	0.4198	−0.0771	0.030*	0.36
C2B	0.3296 (8)	0.4872 (10)	−0.0430 (6)	0.0231 (9)	0.36
C3B	0.3226 (8)	0.5107 (17)	0.0555 (7)	0.0219 (11)	0.36
H3BA	0.3806	0.4652	0.0767	0.026*	0.36
H3BB	0.3374	0.5878	0.0479	0.026*	0.36
C10B	0.4380 (6)	0.5083 (6)	−0.1393 (5)	0.0311 (9)	0.36
H10D	0.4214	0.5032	−0.1976	0.047*	0.36
H10E	0.4993	0.4546	−0.1353	0.047*	0.36
H10F	0.4643	0.5810	−0.1484	0.047*	0.36
C4	0.20140 (13)	0.48447 (12)	0.13178 (11)	0.0206 (3)	
C5	0.14160 (13)	0.48424 (11)	0.23689 (11)	0.0191 (3)	
C6	0.02849 (14)	0.44463 (11)	0.28549 (12)	0.0205 (3)	
H6A	−0.0147	0.4443	0.3566	0.025*	
C7	−0.02250 (13)	0.40571 (12)	0.23245 (12)	0.0209 (3)	
C8	0.03690 (14)	0.40435 (12)	0.12884 (12)	0.0226 (3)	
H8A	0.0023	0.3769	0.0931	0.027*	
C9	0.14855 (14)	0.44441 (12)	0.07904 (11)	0.0224 (3)	
C11	0.19072 (13)	0.52333 (12)	0.29924 (11)	0.0194 (3)	
C12	0.25058 (13)	0.61938 (12)	0.26471 (12)	0.0218 (3)	
H12A	0.2629	0.6604	0.1989	0.026*	
C13	0.29250 (14)	0.65589 (13)	0.32554 (13)	0.0253 (3)	
H13A	0.3328	0.7217	0.3014	0.030*	
C14	0.27550 (14)	0.59619 (13)	0.42142 (12)	0.0259 (3)	
H14A	0.3040	0.6212	0.4630	0.031*	
C15	0.21710 (14)	0.50040 (13)	0.45646 (12)	0.0261 (3)	
H15A	0.2063	0.4590	0.5218	0.031*	
C16	0.17419 (14)	0.46467 (13)	0.39630 (12)	0.0230 (3)	
H16A	0.1329	0.3993	0.4214	0.028*	
C17	−0.23058 (14)	0.45087 (12)	0.30246 (12)	0.0222 (3)	
C18	−0.20467 (14)	0.56058 (12)	0.26732 (12)	0.0239 (3)	
H18A	−0.1254	0.5810	0.2324	0.029*	
C19	−0.29344 (15)	0.63977 (14)	0.28299 (14)	0.0302 (4)	
H19A	−0.2743	0.7137	0.2574	0.036*	
C20	−0.40919 (16)	0.61252 (15)	0.33534 (15)	0.0366 (4)	
H20A	−0.4696	0.6672	0.3472	0.044*	
C21	−0.43612 (15)	0.50402 (15)	0.37037 (15)	0.0349 (4)	
H21A	−0.5155	0.4843	0.4072	0.042*	
C22	−0.34799 (14)	0.42418 (13)	0.35195 (13)	0.0254 (3)	
C23	−0.26889 (14)	0.22643 (12)	0.41132 (12)	0.0241 (3)	
C24	−0.28651 (16)	0.13143 (13)	0.48368 (12)	0.0293 (4)	
H24A	−0.3632	0.1054	0.5227	0.035*	
C25	−0.19306 (17)	0.07471 (14)	0.49914 (13)	0.0339 (4)	
H25A	−0.2049	0.0096	0.5482	0.041*	
C26	−0.08275 (17)	0.11414 (14)	0.44232 (13)	0.0329 (4)	
H26A	−0.0178	0.0744	0.4509	0.039*	
C27	−0.06447 (15)	0.21071 (13)	0.37273 (13)	0.0269 (3)	
H27A	0.0123	0.2371	0.3355	0.032*	
C28	−0.15795 (14)	0.26952 (12)	0.35684 (11)	0.0212 (3)	
S1'	0.52340 (4)	0.90861 (4)	0.22623 (3)	0.03033 (11)	
N1'	0.29876 (11)	0.95936 (10)	0.19830 (10)	0.0224 (3)	
C1'	−0.1142 (6)	0.8341 (9)	0.3848 (8)	0.0280 (9)	0.57
H1'A	−0.1106	0.7772	0.4375	0.034*	0.57
C2'	−0.2113 (5)	0.8666 (9)	0.3686 (9)	0.0234 (17)	0.57
C3'	−0.1847 (4)	0.9641 (10)	0.2835 (9)	0.0274 (7)	0.57
H3'A	−0.2060	0.9525	0.2290	0.033*	0.57
H3'B	−0.2272	1.0298	0.3096	0.033*	0.57
C10'	−0.3346 (6)	0.8357 (13)	0.4355 (11)	0.0367 (7)	0.57
H10G	−0.3351	0.7676	0.4826	0.055*	0.57
H10H	−0.3739	0.8264	0.3931	0.055*	0.57
H10I	−0.3758	0.8928	0.4749	0.055*	0.57
C3"	−0.1803 (5)	0.9560 (14)	0.2818 (12)	0.0274 (7)	0.43
H3"C	−0.2280	0.9889	0.2472	0.033*	0.43
C2"	−0.2181 (6)	0.8864 (14)	0.3722 (13)	0.0234 (17)	0.43
C1"	−0.1148 (8)	0.8372 (13)	0.3937 (10)	0.0280 (9)	0.43
H1"A	−0.1235	0.8469	0.4616	0.034*	0.43
H1"B	−0.1046	0.7590	0.3909	0.034*	0.43
C10"	−0.3371 (7)	0.8421 (17)	0.4344 (15)	0.0367 (7)	0.43
H10J	−0.3880	0.8703	0.3989	0.055*	0.43
H10K	−0.3699	0.8640	0.5014	0.055*	0.43
H10L	−0.3317	0.7629	0.4437	0.055*	0.43
C4'	−0.05486 (13)	0.97368 (13)	0.24479 (12)	0.0231 (3)	
C5'	0.02241 (13)	1.04173 (12)	0.16016 (12)	0.0218 (3)	
C6'	0.14048 (14)	1.03393 (12)	0.14479 (12)	0.0230 (3)	
H6'A	0.1954	1.0785	0.0875	0.028*	
C7'	0.17846 (14)	0.96234 (12)	0.21175 (12)	0.0225 (3)	
C8'	0.10177 (14)	0.89287 (12)	0.29435 (12)	0.0239 (3)	
H8'A	0.1284	0.8427	0.3390	0.029*	
C9'	−0.01475 (13)	0.89899 (12)	0.30968 (11)	0.0239 (3)	
C11'	−0.01489 (14)	1.11879 (12)	0.08630 (12)	0.0229 (3)	
C12'	−0.11953 (15)	1.17980 (13)	0.11959 (14)	0.0283 (4)	
H12B	−0.1673	1.1736	0.1907	0.034*	
C13'	−0.15425 (17)	1.24937 (14)	0.04968 (15)	0.0360 (4)	
H13B	−0.2259	1.2900	0.0732	0.043*	
C14'	−0.08514 (17)	1.25996 (14)	−0.05407 (15)	0.0368 (4)	
H14B	−0.1095	1.3074	−0.1017	0.044*	
C15'	0.01959 (17)	1.20117 (13)	−0.08826 (14)	0.0319 (4)	
H15B	0.0677	1.2087	−0.1594	0.038*	
C16'	0.05434 (15)	1.13104 (13)	−0.01829 (13)	0.0269 (4)	
H16B	0.1263	1.0909	−0.0422	0.032*	
C17'	0.33336 (13)	1.04156 (12)	0.23036 (11)	0.0204 (3)	
C18'	0.26413 (14)	1.13481 (13)	0.25307 (12)	0.0249 (3)	
H18B	0.1917	1.1438	0.2468	0.030*	
C19'	0.29908 (16)	1.21455 (13)	0.28463 (12)	0.0288 (4)	
H19B	0.2502	1.2772	0.3003	0.035*	
C20'	0.40464 (16)	1.20386 (14)	0.29361 (13)	0.0316 (4)	
H20B	0.4289	1.2590	0.3146	0.038*	
C21'	0.47437 (15)	1.11155 (14)	0.27160 (12)	0.0291 (4)	
H21B	0.5473	1.1039	0.2769	0.035*	
C22'	0.43907 (13)	1.03023 (13)	0.24200 (12)	0.0231 (3)	
C23'	0.49271 (14)	0.87210 (13)	0.13113 (12)	0.0251 (3)	
C24'	0.57445 (16)	0.80923 (14)	0.06501 (13)	0.0333 (4)	
H24B	0.6490	0.7934	0.0667	0.040*	
C25'	0.54849 (17)	0.76935 (14)	−0.00342 (13)	0.0363 (4)	
H25B	0.6040	0.7249	−0.0474	0.044*	
C26'	0.44052 (17)	0.79525 (13)	−0.00680 (13)	0.0341 (4)	
H26B	0.4223	0.7686	−0.0539	0.041*	
C27'	0.35867 (15)	0.85936 (13)	0.05747 (12)	0.0275 (4)	
H27B	0.2855	0.8772	0.0532	0.033*	
C28'	0.38264 (14)	0.89814 (12)	0.12854 (12)	0.0226 (3)	

### Atomic displacement parameters (Å^2^)

**Table:**

	*U*^11^	*U*^22^	*U*^33^	*U*^12^	*U*^13^	*U*^23^
S1	0.0311 (2)	0.0292 (2)	0.0475 (3)	−0.00855 (18)	−0.0236 (2)	−0.00198 (19)
N1	0.0246 (7)	0.0195 (6)	0.0234 (7)	−0.0014 (5)	−0.0139 (6)	−0.0025 (5)
C3A	0.0209 (9)	0.023 (3)	0.0244 (8)	0.0050 (12)	−0.0120 (7)	−0.0066 (9)
C2A	0.0276 (10)	0.024 (3)	0.0216 (10)	0.0081 (13)	−0.0133 (9)	−0.0097 (11)
C1A	0.0295 (9)	0.025 (3)	0.0244 (9)	0.0058 (10)	−0.0153 (8)	−0.0097 (11)
C10A	0.0300 (10)	0.039 (3)	0.0222 (10)	0.0123 (16)	−0.0091 (8)	−0.0133 (14)
C1B	0.0295 (9)	0.025 (3)	0.0244 (9)	0.0058 (10)	−0.0153 (8)	−0.0097 (11)
C2B	0.0276 (10)	0.024 (3)	0.0216 (10)	0.0081 (13)	−0.0133 (9)	−0.0097 (11)
C3B	0.0209 (9)	0.023 (3)	0.0244 (8)	0.0050 (12)	−0.0120 (7)	−0.0066 (9)
C10B	0.0300 (10)	0.039 (3)	0.0222 (10)	0.0123 (16)	−0.0091 (8)	−0.0133 (14)
C4	0.0241 (8)	0.0189 (7)	0.0235 (8)	0.0047 (6)	−0.0153 (7)	−0.0045 (6)
C5	0.0254 (8)	0.0150 (7)	0.0215 (8)	0.0031 (6)	−0.0151 (7)	−0.0034 (6)
C6	0.0280 (8)	0.0175 (7)	0.0191 (7)	0.0012 (6)	−0.0135 (7)	−0.0028 (6)
C7	0.0253 (8)	0.0160 (7)	0.0239 (8)	−0.0002 (6)	−0.0139 (7)	−0.0013 (6)
C8	0.0302 (8)	0.0213 (8)	0.0233 (8)	0.0012 (6)	−0.0174 (7)	−0.0057 (6)
C9	0.0294 (8)	0.0210 (7)	0.0217 (8)	0.0061 (6)	−0.0157 (7)	−0.0068 (6)
C11	0.0197 (7)	0.0218 (7)	0.0199 (7)	0.0043 (6)	−0.0110 (6)	−0.0067 (6)
C12	0.0239 (8)	0.0227 (8)	0.0213 (8)	0.0014 (6)	−0.0123 (7)	−0.0041 (6)
C13	0.0243 (8)	0.0250 (8)	0.0313 (9)	−0.0009 (6)	−0.0141 (7)	−0.0084 (7)
C14	0.0270 (8)	0.0322 (9)	0.0275 (8)	0.0057 (7)	−0.0178 (7)	−0.0136 (7)
C15	0.0320 (9)	0.0300 (9)	0.0203 (8)	0.0060 (7)	−0.0153 (7)	−0.0072 (7)
C16	0.0281 (8)	0.0215 (8)	0.0216 (8)	0.0018 (6)	−0.0123 (7)	−0.0058 (6)
C17	0.0283 (8)	0.0231 (8)	0.0224 (8)	0.0009 (6)	−0.0176 (7)	−0.0047 (6)
C18	0.0281 (8)	0.0233 (8)	0.0260 (8)	−0.0015 (7)	−0.0174 (7)	−0.0025 (6)
C19	0.0373 (10)	0.0228 (8)	0.0377 (10)	0.0017 (7)	−0.0238 (8)	−0.0037 (7)
C20	0.0335 (10)	0.0311 (10)	0.0543 (12)	0.0086 (8)	−0.0280 (9)	−0.0106 (9)
C21	0.0259 (9)	0.0397 (10)	0.0446 (11)	0.0006 (8)	−0.0199 (8)	−0.0082 (8)
C22	0.0285 (8)	0.0254 (8)	0.0299 (9)	−0.0031 (7)	−0.0197 (7)	−0.0027 (7)
C23	0.0320 (9)	0.0224 (8)	0.0232 (8)	−0.0015 (7)	−0.0147 (7)	−0.0071 (6)
C24	0.0403 (10)	0.0238 (8)	0.0222 (8)	−0.0071 (7)	−0.0101 (8)	−0.0035 (7)
C25	0.0537 (12)	0.0218 (8)	0.0239 (9)	−0.0022 (8)	−0.0160 (9)	0.0007 (7)
C26	0.0423 (11)	0.0258 (9)	0.0331 (10)	0.0061 (8)	−0.0207 (9)	−0.0034 (7)
C27	0.0306 (9)	0.0228 (8)	0.0288 (9)	0.0018 (7)	−0.0143 (8)	−0.0051 (7)
C28	0.0299 (8)	0.0187 (7)	0.0188 (7)	−0.0015 (6)	−0.0129 (7)	−0.0045 (6)
S1'	0.0272 (2)	0.0343 (2)	0.0333 (2)	0.00648 (18)	−0.01859 (19)	−0.00491 (18)
N1'	0.0209 (6)	0.0239 (7)	0.0257 (7)	0.0022 (5)	−0.0122 (6)	−0.0071 (5)
C1'	0.0333 (9)	0.0284 (11)	0.0237 (15)	−0.0090 (8)	−0.0120 (9)	−0.0028 (12)
C2'	0.0287 (10)	0.019 (4)	0.0246 (11)	−0.0039 (16)	−0.0081 (10)	−0.013 (2)
C3'	0.0242 (9)	0.0370 (17)	0.0240 (9)	−0.0053 (9)	−0.0111 (8)	−0.0068 (10)
C10'	0.0325 (10)	0.048 (2)	0.0277 (10)	−0.0138 (10)	−0.0077 (8)	−0.0067 (10)
C3"	0.0242 (9)	0.0370 (17)	0.0240 (9)	−0.0053 (9)	−0.0111 (8)	−0.0068 (10)
C2"	0.0287 (10)	0.019 (4)	0.0246 (11)	−0.0039 (16)	−0.0081 (10)	−0.013 (2)
C1"	0.0333 (9)	0.0284 (11)	0.0237 (15)	−0.0090 (8)	−0.0120 (9)	−0.0028 (12)
C10"	0.0325 (10)	0.048 (2)	0.0277 (10)	−0.0138 (10)	−0.0077 (8)	−0.0067 (10)
C4'	0.0243 (8)	0.0273 (8)	0.0218 (8)	−0.0023 (6)	−0.0118 (7)	−0.0068 (6)
C5'	0.0251 (8)	0.0218 (8)	0.0235 (8)	−0.0016 (6)	−0.0137 (7)	−0.0058 (6)
C6'	0.0245 (8)	0.0234 (8)	0.0223 (8)	−0.0042 (6)	−0.0111 (7)	−0.0019 (6)
C7'	0.0240 (8)	0.0229 (8)	0.0249 (8)	−0.0004 (6)	−0.0127 (7)	−0.0077 (6)
C8'	0.0298 (9)	0.0218 (8)	0.0233 (8)	0.0005 (7)	−0.0146 (7)	−0.0032 (6)
C9'	0.0281 (8)	0.0241 (8)	0.0210 (8)	−0.0048 (7)	−0.0106 (7)	−0.0039 (6)
C11'	0.0266 (8)	0.0215 (8)	0.0275 (8)	−0.0038 (6)	−0.0173 (7)	−0.0035 (6)
C12'	0.0301 (9)	0.0283 (9)	0.0344 (9)	0.0000 (7)	−0.0187 (8)	−0.0104 (7)
C13'	0.0390 (10)	0.0280 (9)	0.0530 (12)	0.0066 (8)	−0.0310 (10)	−0.0102 (8)
C14'	0.0543 (12)	0.0235 (9)	0.0482 (12)	−0.0013 (8)	−0.0393 (10)	0.0009 (8)
C15'	0.0462 (11)	0.0235 (8)	0.0322 (9)	−0.0066 (8)	−0.0235 (9)	0.0009 (7)
C16'	0.0327 (9)	0.0229 (8)	0.0287 (9)	−0.0021 (7)	−0.0170 (8)	−0.0024 (7)
C17'	0.0222 (8)	0.0215 (8)	0.0177 (7)	−0.0021 (6)	−0.0093 (6)	−0.0008 (6)
C18'	0.0276 (8)	0.0252 (8)	0.0231 (8)	0.0018 (7)	−0.0124 (7)	−0.0043 (6)
C19'	0.0386 (10)	0.0253 (8)	0.0241 (8)	0.0009 (7)	−0.0142 (8)	−0.0060 (7)
C20'	0.0441 (11)	0.0300 (9)	0.0269 (9)	−0.0099 (8)	−0.0187 (8)	−0.0039 (7)
C21'	0.0293 (9)	0.0364 (10)	0.0265 (9)	−0.0075 (7)	−0.0168 (8)	−0.0007 (7)
C22'	0.0228 (8)	0.0269 (8)	0.0196 (8)	−0.0012 (6)	−0.0102 (7)	−0.0008 (6)
C23'	0.0266 (8)	0.0228 (8)	0.0224 (8)	−0.0009 (6)	−0.0089 (7)	0.0004 (6)
C24'	0.0298 (9)	0.0283 (9)	0.0302 (9)	0.0027 (7)	−0.0046 (8)	−0.0002 (7)
C25'	0.0443 (11)	0.0242 (9)	0.0248 (9)	0.0006 (8)	−0.0005 (8)	−0.0036 (7)
C26'	0.0534 (12)	0.0229 (8)	0.0227 (9)	−0.0068 (8)	−0.0122 (8)	−0.0015 (7)
C27'	0.0359 (9)	0.0226 (8)	0.0237 (8)	−0.0035 (7)	−0.0128 (8)	−0.0007 (6)
C28'	0.0274 (8)	0.0173 (7)	0.0203 (8)	−0.0020 (6)	−0.0090 (7)	0.0008 (6)

### Geometric parameters (Å, °)

**Table:**

S1—C23	1.7621 (16)	S1'—C23'	1.7617 (17)
S1—C22	1.7643 (16)	S1'—C22'	1.7624 (16)
N1—C28	1.4206 (19)	N1'—C28'	1.412 (2)
N1—C17	1.4217 (19)	N1'—C17'	1.4132 (19)
N1—C7	1.442 (2)	N1'—C7'	1.4453 (19)
C3A—C2A	1.351 (6)	C1'—C2'	1.348 (6)
C3A—C4	1.478 (3)	C1'—C9'	1.467 (4)
C3A—H3AA	0.9500	C1'—H1'A	0.9500
C2A—C10A	1.492 (3)	C2'—C10'	1.493 (3)
C2A—C1A	1.502 (6)	C2'—C3'	1.510 (7)
C1A—C9	1.507 (3)	C3'—C4'	1.503 (4)
C1A—H1AA	0.9900	C3'—H3'A	0.9900
C1A—H1AB	0.9900	C3'—H3'B	0.9900
C10A—H10A	0.9800	C10'—H10G	0.9800
C10A—H10B	0.9800	C10'—H10H	0.9800
C10A—H10C	0.9800	C10'—H10I	0.9800
C1B—C2B	1.345 (7)	C3"—C2"	1.352 (7)
C1B—C9	1.461 (5)	C3"—C4'	1.471 (4)
C1B—H1BA	0.9500	C3"—H3"C	0.9500
C2B—C10B	1.493 (3)	C2"—C10"	1.493 (3)
C2B—C3B	1.504 (8)	C2"—C1"	1.503 (8)
C3B—C4	1.489 (5)	C1"—C9'	1.494 (4)
C3B—H3BA	0.9900	C1"—H1"A	0.9900
C3B—H3BB	0.9900	C1"—H1"B	0.9900
C10B—H10D	0.9800	C10"—H10J	0.9800
C10B—H10E	0.9800	C10"—H10K	0.9800
C10B—H10F	0.9800	C10"—H10L	0.9800
C4—C5	1.401 (2)	C4'—C5'	1.397 (2)
C4—C9	1.414 (2)	C4'—C9'	1.402 (2)
C5—C6	1.400 (2)	C5'—C6'	1.406 (2)
C5—C11	1.493 (2)	C5'—C11'	1.489 (2)
C6—C7	1.393 (2)	C6'—C7'	1.389 (2)
C6—H6A	0.9500	C6'—H6'A	0.9500
C7—C8	1.385 (2)	C7'—C8'	1.390 (2)
C8—C9	1.386 (2)	C8'—C9'	1.388 (2)
C8—H8A	0.9500	C8'—H8'A	0.9500
C11—C12	1.393 (2)	C11'—C16'	1.392 (2)
C11—C16	1.398 (2)	C11'—C12'	1.397 (2)
C12—C13	1.392 (2)	C12'—C13'	1.386 (2)
C12—H12A	0.9500	C12'—H12B	0.9500
C13—C14	1.387 (2)	C13'—C14'	1.383 (3)
C13—H13A	0.9500	C13'—H13B	0.9500
C14—C15	1.381 (2)	C14'—C15'	1.384 (3)
C14—H14A	0.9500	C14'—H14B	0.9500
C15—C16	1.387 (2)	C15'—C16'	1.390 (2)
C15—H15A	0.9500	C15'—H15B	0.9500
C16—H16A	0.9500	C16'—H16B	0.9500
C17—C22	1.395 (2)	C17'—C18'	1.394 (2)
C17—C18	1.401 (2)	C17'—C22'	1.405 (2)
C18—C19	1.387 (2)	C18'—C19'	1.385 (2)
C18—H18A	0.9500	C18'—H18B	0.9500
C19—C20	1.380 (3)	C19'—C20'	1.384 (2)
C19—H19A	0.9500	C19'—H19B	0.9500
C20—C21	1.389 (3)	C20'—C21'	1.385 (2)
C20—H20A	0.9500	C20'—H20B	0.9500
C21—C22	1.387 (2)	C21'—C22'	1.386 (2)
C21—H21A	0.9500	C21'—H21B	0.9500
C23—C24	1.392 (2)	C23'—C24'	1.388 (2)
C23—C28	1.396 (2)	C23'—C28'	1.406 (2)
C24—C25	1.384 (2)	C24'—C25'	1.384 (3)
C24—H24A	0.9500	C24'—H24B	0.9500
C25—C26	1.375 (3)	C25'—C26'	1.384 (3)
C25—H25A	0.9500	C25'—H25B	0.9500
C26—C27	1.386 (2)	C26'—C27'	1.385 (2)
C26—H26A	0.9500	C26'—H26B	0.9500
C27—C28	1.397 (2)	C27'—C28'	1.397 (2)
C27—H27A	0.9500	C27'—H27B	0.9500
			
C23—S1—C22	99.26 (7)	C23—C28—C27	117.81 (14)
C28—N1—C17	120.20 (13)	C23—C28—N1	120.99 (14)
C28—N1—C7	116.80 (12)	C27—C28—N1	121.19 (14)
C17—N1—C7	115.94 (12)	C23'—S1'—C22'	99.80 (7)
C2A—C3A—C4	109.6 (3)	C28'—N1'—C17'	120.76 (13)
C2A—C3A—H3AA	125.2	C28'—N1'—C7'	118.85 (12)
C4—C3A—H3AA	125.2	C17'—N1'—C7'	117.57 (12)
C3A—C2A—C10A	128.7 (4)	C2'—C1'—C9'	110.6 (4)
C3A—C2A—C1A	109.7 (2)	C2'—C1'—H1'A	124.7
C10A—C2A—C1A	121.6 (4)	C9'—C1'—H1'A	124.7
C2A—C1A—C9	104.8 (3)	C1'—C2'—C10'	128.5 (5)
C2A—C1A—H1AA	110.8	C1'—C2'—C3'	109.2 (3)
C9—C1A—H1AA	110.8	C10'—C2'—C3'	120.7 (5)
C2A—C1A—H1AB	110.8	C4'—C3'—C2'	103.4 (4)
C9—C1A—H1AB	110.8	C4'—C3'—H3'A	111.1
H1AA—C1A—H1AB	108.9	C2'—C3'—H3'A	111.1
C2B—C1B—C9	107.7 (5)	C4'—C3'—H3'B	111.1
C2B—C1B—H1BA	126.1	C2'—C3'—H3'B	111.1
C9—C1B—H1BA	126.1	H3'A—C3'—H3'B	109.1
C1B—C2B—C10B	128.7 (6)	C2"—C3"—C4'	110.2 (5)
C1B—C2B—C3B	110.3 (3)	C2"—C3"—H3"C	124.9
C10B—C2B—C3B	121.0 (6)	C4'—C3"—H3"C	124.9
C4—C3B—C2B	105.2 (4)	C3"—C2"—C10"	128.3 (7)
C4—C3B—H3BA	110.7	C3"—C2"—C1"	109.4 (3)
C2B—C3B—H3BA	110.7	C10"—C2"—C1"	121.1 (6)
C4—C3B—H3BB	110.7	C9'—C1"—C2"	103.1 (5)
C2B—C3B—H3BB	110.7	C9'—C1"—H1"A	111.2
H3BA—C3B—H3BB	108.8	C2"—C1"—H1"A	111.2
C2B—C10B—H10D	109.5	C9'—C1"—H1"B	111.2
C2B—C10B—H10E	109.5	C2"—C1"—H1"B	111.2
H10D—C10B—H10E	109.5	H1"A—C1"—H1"B	109.1
C2B—C10B—H10F	109.5	C5'—C4'—C9'	120.86 (14)
H10D—C10B—H10F	109.5	C5'—C4'—C3"	132.4 (5)
H10E—C10B—H10F	109.5	C9'—C4'—C3"	106.6 (5)
C5—C4—C9	120.40 (13)	C5'—C4'—C3'	130.3 (4)
C5—C4—C3A	130.8 (3)	C9'—C4'—C3'	108.8 (4)
C9—C4—C3A	108.7 (2)	C4'—C5'—C6'	117.28 (14)
C5—C4—C3B	133.7 (4)	C4'—C5'—C11'	122.90 (14)
C9—C4—C3B	105.6 (4)	C6'—C5'—C11'	119.81 (14)
C6—C5—C4	117.16 (13)	C7'—C6'—C5'	121.33 (15)
C6—C5—C11	118.59 (13)	C7'—C6'—H6'A	119.3
C4—C5—C11	124.26 (13)	C5'—C6'—H6'A	119.3
C7—C6—C5	121.77 (14)	C6'—C7'—C8'	121.13 (14)
C7—C6—H6A	119.1	C6'—C7'—N1'	120.77 (14)
C5—C6—H6A	119.1	C8'—C7'—N1'	118.10 (13)
C8—C7—C6	121.28 (14)	C9'—C8'—C7'	118.05 (14)
C8—C7—N1	119.44 (13)	C9'—C8'—H8'A	121.0
C6—C7—N1	119.26 (14)	C7'—C8'—H8'A	121.0
C7—C8—C9	117.84 (14)	C8'—C9'—C4'	121.29 (14)
C7—C8—H8A	121.1	C8'—C9'—C1'	131.1 (4)
C9—C8—H8A	121.1	C4'—C9'—C1'	107.5 (4)
C8—C9—C4	121.55 (13)	C8'—C9'—C1"	128.8 (5)
C8—C9—C1B	127.3 (4)	C4'—C9'—C1"	109.9 (5)
C4—C9—C1B	111.0 (4)	C16'—C11'—C12'	118.44 (14)
C8—C9—C1A	131.1 (3)	C16'—C11'—C5'	120.28 (14)
C4—C9—C1A	107.2 (2)	C12'—C11'—C5'	121.27 (15)
C12—C11—C16	118.30 (14)	C13'—C12'—C11'	120.54 (17)
C12—C11—C5	121.74 (13)	C13'—C12'—H12B	119.7
C16—C11—C5	119.94 (13)	C11'—C12'—H12B	119.7
C13—C12—C11	120.71 (14)	C14'—C13'—C12'	120.39 (17)
C13—C12—H12A	119.6	C14'—C13'—H13B	119.8
C11—C12—H12A	119.6	C12'—C13'—H13B	119.8
C14—C13—C12	120.02 (15)	C13'—C14'—C15'	119.79 (16)
C14—C13—H13A	120.0	C13'—C14'—H14B	120.1
C12—C13—H13A	120.0	C15'—C14'—H14B	120.1
C15—C14—C13	119.98 (14)	C14'—C15'—C16'	119.91 (17)
C15—C14—H14A	120.0	C14'—C15'—H15B	120.0
C13—C14—H14A	120.0	C16'—C15'—H15B	120.0
C14—C15—C16	119.97 (15)	C15'—C16'—C11'	120.92 (16)
C14—C15—H15A	120.0	C15'—C16'—H16B	119.5
C16—C15—H15A	120.0	C11'—C16'—H16B	119.5
C15—C16—C11	121.01 (15)	C18'—C17'—C22'	117.86 (14)
C15—C16—H16A	119.5	C18'—C17'—N1'	121.80 (13)
C11—C16—H16A	119.5	C22'—C17'—N1'	120.33 (14)
C22—C17—C18	117.88 (14)	C19'—C18'—C17'	121.14 (15)
C22—C17—N1	120.76 (14)	C19'—C18'—H18B	119.4
C18—C17—N1	121.34 (14)	C17'—C18'—H18B	119.4
C19—C18—C17	120.71 (15)	C20'—C19'—C18'	120.61 (16)
C19—C18—H18A	119.6	C20'—C19'—H19B	119.7
C17—C18—H18A	119.6	C18'—C19'—H19B	119.7
C20—C19—C18	120.84 (16)	C19'—C20'—C21'	118.99 (15)
C20—C19—H19A	119.6	C19'—C20'—H20B	120.5
C18—C19—H19A	119.6	C21'—C20'—H20B	120.5
C19—C20—C21	119.03 (16)	C20'—C21'—C22'	120.84 (15)
C19—C20—H20A	120.5	C20'—C21'—H21B	119.6
C21—C20—H20A	120.5	C22'—C21'—H21B	119.6
C22—C21—C20	120.46 (17)	C21'—C22'—C17'	120.51 (15)
C22—C21—H21A	119.8	C21'—C22'—S1'	119.53 (12)
C20—C21—H21A	119.8	C17'—C22'—S1'	119.85 (12)
C21—C22—C17	120.98 (15)	C24'—C23'—C28'	120.54 (16)
C21—C22—S1	118.54 (13)	C24'—C23'—S1'	119.08 (13)
C17—C22—S1	120.31 (12)	C28'—C23'—S1'	120.12 (12)
C24—C23—C28	120.85 (15)	C25'—C24'—C23'	120.71 (17)
C24—C23—S1	118.92 (13)	C25'—C24'—H24B	119.6
C28—C23—S1	120.13 (12)	C23'—C24'—H24B	119.6
C25—C24—C23	120.47 (16)	C24'—C25'—C26'	119.03 (17)
C25—C24—H24A	119.8	C24'—C25'—H25B	120.5
C23—C24—H24A	119.8	C26'—C25'—H25B	120.5
C26—C25—C24	118.89 (15)	C25'—C26'—C27'	120.99 (17)
C26—C25—H25A	120.6	C25'—C26'—H26B	119.5
C24—C25—H25A	120.6	C27'—C26'—H26B	119.5
C25—C26—C27	121.25 (16)	C26'—C27'—C28'	120.61 (16)
C25—C26—H26A	119.4	C26'—C27'—H27B	119.7
C27—C26—H26A	119.4	C28'—C27'—H27B	119.7
C26—C27—C28	120.59 (16)	C27'—C28'—C23'	118.09 (15)
C26—C27—H27A	119.7	C27'—C28'—N1'	121.81 (14)
C28—C27—H27A	119.7	C23'—C28'—N1'	120.08 (14)
			
C4—C3A—C2A—C10A	178.3 (6)	C9'—C1'—C2'—C10'	−169.4 (14)
C4—C3A—C2A—C1A	1.0 (10)	C9'—C1'—C2'—C3'	−4.3 (16)
C3A—C2A—C1A—C9	−1.0 (10)	C1'—C2'—C3'—C4'	6.9 (14)
C10A—C2A—C1A—C9	−178.6 (5)	C10'—C2'—C3'—C4'	173.4 (13)
C9—C1B—C2B—C10B	−178.0 (12)	C4'—C3"—C2"—C10"	−177.0 (19)
C9—C1B—C2B—C3B	4(2)	C4'—C3"—C2"—C1"	−10 (2)
C1B—C2B—C3B—C4	−4(2)	C3"—C2"—C1"—C9'	7(2)
C10B—C2B—C3B—C4	177.5 (11)	C10"—C2"—C1"—C9'	175.3 (17)
C2A—C3A—C4—C5	−177.1 (4)	C2"—C3"—C4'—C5'	−175.7 (10)
C2A—C3A—C4—C9	−0.5 (8)	C2"—C3"—C4'—C9'	8.5 (16)
C2A—C3A—C4—C3B	69 (5)	C2"—C3"—C4'—C3'	−114 (15)
C2B—C3B—C4—C5	176.8 (7)	C2'—C3'—C4'—C5'	172.9 (6)
C2B—C3B—C4—C9	2.7 (15)	C2'—C3'—C4'—C9'	−7.1 (10)
C2B—C3B—C4—C3A	−111 (6)	C2'—C3'—C4'—C3"	51 (14)
C9—C4—C5—C6	−0.9 (2)	C9'—C4'—C5'—C6'	−1.6 (2)
C3A—C4—C5—C6	175.3 (6)	C3"—C4'—C5'—C6'	−176.9 (11)
C3B—C4—C5—C6	−174.3 (12)	C3'—C4'—C5'—C6'	178.3 (8)
C9—C4—C5—C11	179.11 (13)	C9'—C4'—C5'—C11'	177.11 (14)
C3A—C4—C5—C11	−4.6 (6)	C3"—C4'—C5'—C11'	1.9 (11)
C3B—C4—C5—C11	5.7 (12)	C3'—C4'—C5'—C11'	−3.0 (8)
C4—C5—C6—C7	0.8 (2)	C4'—C5'—C6'—C7'	−0.7 (2)
C11—C5—C6—C7	−179.27 (13)	C11'—C5'—C6'—C7'	−179.51 (14)
C5—C6—C7—C8	0.1 (2)	C5'—C6'—C7'—C8'	2.4 (2)
C5—C6—C7—N1	−178.26 (13)	C5'—C6'—C7'—N1'	−176.53 (14)
C28—N1—C7—C8	109.96 (16)	C28'—N1'—C7'—C6'	−83.26 (18)
C17—N1—C7—C8	−99.29 (16)	C17'—N1'—C7'—C6'	77.90 (18)
C28—N1—C7—C6	−71.64 (17)	C28'—N1'—C7'—C8'	97.75 (17)
C17—N1—C7—C6	79.11 (17)	C17'—N1'—C7'—C8'	−101.09 (16)
C6—C7—C8—C9	−0.8 (2)	C6'—C7'—C8'—C9'	−1.7 (2)
N1—C7—C8—C9	177.53 (13)	N1'—C7'—C8'—C9'	177.33 (14)
C7—C8—C9—C4	0.7 (2)	C7'—C8'—C9'—C4'	−0.7 (2)
C7—C8—C9—C1B	175.9 (11)	C7'—C8'—C9'—C1'	176.1 (8)
C7—C8—C9—C1A	−175.1 (6)	C7'—C8'—C9'—C1"	−177.6 (11)
C5—C4—C9—C8	0.2 (2)	C5'—C4'—C9'—C8'	2.4 (2)
C3A—C4—C9—C8	−176.8 (4)	C3"—C4'—C9'—C8'	178.7 (8)
C3B—C4—C9—C8	175.3 (9)	C3'—C4'—C9'—C8'	−177.5 (6)
C5—C4—C9—C1B	−175.7 (9)	C5'—C4'—C9'—C1'	−175.1 (6)
C3A—C4—C9—C1B	7.3 (10)	C3"—C4'—C9'—C1'	1.2 (11)
C3B—C4—C9—C1B	−0.6 (13)	C3'—C4'—C9'—C1'	4.9 (9)
C5—C4—C9—C1A	176.9 (5)	C5'—C4'—C9'—C1"	179.8 (9)
C3A—C4—C9—C1A	−0.2 (6)	C3"—C4'—C9'—C1"	−3.8 (12)
C3B—C4—C9—C1A	−8.1 (10)	C3'—C4'—C9'—C1"	−0.1 (11)
C2B—C1B—C9—C8	−177.5 (9)	C2'—C1'—C9'—C8'	−177.6 (8)
C2B—C1B—C9—C4	−1.9 (17)	C2'—C1'—C9'—C4'	−0.4 (13)
C2B—C1B—C9—C1A	61 (5)	C2'—C1'—C9'—C1"	117 (12)
C2A—C1A—C9—C8	176.9 (4)	C2"—C1"—C9'—C8'	175.6 (9)
C2A—C1A—C9—C4	0.7 (8)	C2"—C1"—C9'—C4'	−1.6 (15)
C2A—C1A—C9—C1B	−119 (6)	C2"—C1"—C9'—C1'	−66 (10)
C6—C5—C11—C12	−135.67 (15)	C4'—C5'—C11'—C16'	−138.00 (16)
C4—C5—C11—C12	44.3 (2)	C6'—C5'—C11'—C16'	40.7 (2)
C6—C5—C11—C16	42.5 (2)	C4'—C5'—C11'—C12'	41.7 (2)
C4—C5—C11—C16	−137.61 (15)	C6'—C5'—C11'—C12'	−139.62 (16)
C16—C11—C12—C13	−0.1 (2)	C16'—C11'—C12'—C13'	1.1 (2)
C5—C11—C12—C13	178.02 (14)	C5'—C11'—C12'—C13'	−178.54 (15)
C11—C12—C13—C14	0.4 (2)	C11'—C12'—C13'—C14'	−0.5 (3)
C12—C13—C14—C15	0.1 (2)	C12'—C13'—C14'—C15'	−0.3 (3)
C13—C14—C15—C16	−0.8 (2)	C13'—C14'—C15'—C16'	0.6 (3)
C14—C15—C16—C11	1.1 (2)	C14'—C15'—C16'—C11'	0.0 (2)
C12—C11—C16—C15	−0.6 (2)	C12'—C11'—C16'—C15'	−0.9 (2)
C5—C11—C16—C15	−178.78 (14)	C5'—C11'—C16'—C15'	178.79 (15)
C28—N1—C17—C22	−31.4 (2)	C28'—N1'—C17'—C18'	146.99 (15)
C7—N1—C17—C22	178.91 (13)	C7'—N1'—C17'—C18'	−13.8 (2)
C28—N1—C17—C18	150.34 (14)	C28'—N1'—C17'—C22'	−34.1 (2)
C7—N1—C17—C18	0.6 (2)	C7'—N1'—C17'—C22'	165.08 (14)
C22—C17—C18—C19	1.0 (2)	C22'—C17'—C18'—C19'	0.9 (2)
N1—C17—C18—C19	179.33 (14)	N1'—C17'—C18'—C19'	179.85 (14)
C17—C18—C19—C20	1.3 (2)	C17'—C18'—C19'—C20'	0.5 (2)
C18—C19—C20—C21	−1.5 (3)	C18'—C19'—C20'—C21'	−0.7 (2)
C19—C20—C21—C22	−0.8 (3)	C19'—C20'—C21'—C22'	−0.7 (2)
C20—C21—C22—C17	3.2 (3)	C20'—C21'—C22'—C17'	2.2 (2)
C20—C21—C22—S1	−172.20 (14)	C20'—C21'—C22'—S1'	−174.09 (13)
C18—C17—C22—C21	−3.2 (2)	C18'—C17'—C22'—C21'	−2.3 (2)
N1—C17—C22—C21	178.44 (15)	N1'—C17'—C22'—C21'	178.80 (14)
C18—C17—C22—S1	172.04 (11)	C18'—C17'—C22'—S1'	173.97 (12)
N1—C17—C22—S1	−6.3 (2)	N1'—C17'—C22'—S1'	−4.9 (2)
C23—S1—C22—C21	−150.44 (14)	C23'—S1'—C22'—C21'	−150.37 (13)
C23—S1—C22—C17	34.16 (14)	C23'—S1'—C22'—C17'	33.35 (14)
C22—S1—C23—C24	149.48 (13)	C22'—S1'—C23'—C24'	152.87 (13)
C22—S1—C23—C28	−34.10 (14)	C22'—S1'—C23'—C28'	−32.85 (14)
C28—C23—C24—C25	−3.4 (2)	C28'—C23'—C24'—C25'	−1.2 (2)
S1—C23—C24—C25	172.94 (13)	S1'—C23'—C24'—C25'	173.09 (13)
C23—C24—C25—C26	0.4 (3)	C23'—C24'—C25'—C26'	1.6 (3)
C24—C25—C26—C27	2.0 (3)	C24'—C25'—C26'—C27'	−0.5 (3)
C25—C26—C27—C28	−1.4 (3)	C25'—C26'—C27'—C28'	−1.0 (2)
C24—C23—C28—C27	3.9 (2)	C26'—C27'—C28'—C23'	1.4 (2)
S1—C23—C28—C27	−172.41 (12)	C26'—C27'—C28'—N1'	−176.94 (14)
C24—C23—C28—N1	−177.39 (14)	C24'—C23'—C28'—C27'	−0.3 (2)
S1—C23—C28—N1	6.3 (2)	S1'—C23'—C28'—C27'	−174.49 (12)
C26—C27—C28—C23	−1.5 (2)	C24'—C23'—C28'—N1'	178.03 (14)
C26—C27—C28—N1	179.80 (15)	S1'—C23'—C28'—N1'	3.8 (2)
C17—N1—C28—C23	31.4 (2)	C17'—N1'—C28'—C27'	−147.03 (14)
C7—N1—C28—C23	−179.11 (13)	C7'—N1'—C28'—C27'	13.5 (2)
C17—N1—C28—C27	−149.93 (14)	C17'—N1'—C28'—C23'	34.7 (2)
C7—N1—C28—C27	−0.5 (2)	C7'—N1'—C28'—C23'	−164.74 (14)

## References

[bb1] Allen, F. H. (2002). *Acta Cryst.* B**58**, 380–388. 10.1107/s0108768102003890 12037359

[bb2] Allen, F. H., Kennard, O., Watson, D. G., Brammer, L., Orpen, A. G. & Taylor, R. (1987). *J. Chem. Soc. Perkin Trans. 2*, pp. S1–19.

[bb3] Bruker (2005). *APEX2 *and**SAINT** Bruker AXS Inc., Madison, Wisconsin, USA.

[bb4] Sheldrick, G. M. (2008). *Acta Cryst.* A**64**, 112–122. 10.1107/S0108767307043930 18156677

[bb6] Voskoboynikov, A. Z., Lebedev, A. Y., Izmer, V. V., Ryabov, A. N., Nikulin, M. V. & Canish, J. M. (2006). (ExxonMobil Chemical Patents Inc., USA.) PCT Int. Appl. WO 2 005 105 864.

[bb5] Yang, B. H. & Buchwald, S. L. (1999). *J. Organomet. Chem.* **576**, 125–146.

